# Differential affection of the visual information sub-streams in a patient with visual agnosia

**DOI:** 10.3389/fpsyg.2025.1452979

**Published:** 2025-02-12

**Authors:** Kirstin Lederer, Bruno Fimm, Jorn Munzert, Mathias Reiser, Heiko Maurer, Ferdinand Binkofski, Antonello Pellicano

**Affiliations:** ^1^Division for Clinical Cognitive Research, Department of Neurology, University Hospital RWTH Aachen, Aachen, Germany; ^2^Department of Neurology, University Hospital RWTH Aachen, Aachen, Germany; ^3^Institute for Sport Sciences, University of Gießen, Giessen, Germany; ^4^Institute for Neuroscience and Medicine (INM-4), Research Center Jülich GmbH, Jülich-Aachen, Germany; ^5^JARA Brain, Jülich-Aachen, Germany; ^6^Department of Educational Sciences, University of Catania, Catania, Italy

**Keywords:** visual agnosia, V2 lesion, kinematics, grasping, color information, dorsal stream, ventral stream

## Abstract

**Introduction:**

Visual agnosia is a deficit of object recognition addressed to the damage of the ventral stream (VS). The dorsal stream (DS) is usually intact in these patients, and it can be derived from well-preserved reaching and grasping of visually presented objects. In this study, we presented a new case of a visual agnosic patient (AC) with an extensive lesion of the secondary visual cortex.

**Methods:**

We examined the kinematics of his grasping behavior towards common day-to-day objects compared to a healthy control group. Both colored and color-masked objects were presented, and participants were instructed to grasp-then-name and name-then-grasp them.

**Results:**

The agnosic deficit was particularly evident when no color information was available to the patient: Although AC was able to recognize most colored objects with marked delay, his recognition of color-masked objects was very poor. Furthermore, the color-masked condition determined larger impairments in kinematic performance relative to the control group.

**Discussion:**

Results support the view that spared color processing in the VS allows for partial compensation of deficits. Color information is also processed along the DS, contributing to visuomotor transformations.

## 1 Introduction

According to the influential Two Visual Streams model, the processing of visual information beyond primary visual cortex (V1) is task-dependent and facilitated via a *ventral visual stream* (VS) responsible for conscious object *recognition* (i.e., vision for perception), as opposed to a *dorsal visual stream* (DS) that underlies motor *interaction* with objects (i.e., vision for action) (Goodale and Milner, 2013; Goodale and Milner, 1992, 2018; Goodale et al., 1991; Milner and Goodale, 2008; Milner et al., 1991).

The Two Visual Stream model rests on a large number of single-case studies based on a few individuals and predominantly on elaborate examinations of the visual agnosic patient D.F., as an arguably prototypical patient for a VS deficit (Ganel and Goodale, 2019; Himmelbach et al., 2012; James et al., 2003; Karnath et al., 2009).

While the original model postulated a dichotomous concept, the authors later proposed that the two streams interact to enable skilled actions (de Haan et al., 2018; Ganel and Goodale, 2019; Himmelbach et al., 2012; Himmelbach and Karnath, 2005; Karnath et al., 2009; Konen and Kastner, 2008; Milner, 2017; Pisella et al., 2006; Schenk and McIntosh, 2010).

Along this line, evidence has been provided that the VS is responsible for the processing of visual characteristics of objects like shape, color, and texture (Cavina-Pratesi et al., 2010). Further evidence suggests that color and shape are processed in a segregated and parallel fashion within the VS and converge only at higher levels of visual processing (Lafer-Sousa et al., 2016; Tanaka et al., 2001). Crucially, there is also evidence that parts of the DS utilize color information for action programming (Claeys et al., 2004) and contribute to semantic processing (Creem and Proffitt, 2001; Noppeney et al., 2005). Animal studies show that parts of area V4 show strong connections with dorsal stream areas such as DP, VIP LIP, PIP, or MST. This could be the direct link between VS and DS, providing color information to areas processing action, spatial vision, and spatial attention (Roe et al., 2012; Baizer et al., 1991; Ungerleider et al., 2008).

In sum, these works supported the view that the motor interaction with recognizable objects and within a naturalistic setting, beyond the involvement of visuomotor processing, also implies the identification and the functional analysis of target objects (Borchers and Himmelbach, 2012).

Numerous studies put in evidence residual abilities after the damage of the VS. Investigations on patient D.F. (Ganel and Goodale, 2019), as well as on patient H.J.A. (Chainay and Humphreys, 2001) demonstrated part-based recognition strategies that depended mainly on color and texture features of real-life objects, thus drawing upon their remaining VS function (James et al., 2003). This allowed, for example, D.F. to reach and grasp familiar objects according to their stable affordances (Borghi and Riggio, 2015; Pellicano et al., 2011) as long as the objects were presented in a typical perspective (Carey et al., 1996). Notably, familiarity with real-life objects improves grasping performance in healthy participants (Borchers and Himmelbach, 2012). Even artificial learned associations between the size and the coloring or surface patterns of blocks facilitated grasping performance in healthy participants (Haffenden and Goodale, 2000, 2002). The authors concluded that “[I]ncorporating learned information about object size would reduce the computational demands on the visuomotor system and could allow for efficient and accurate movements directed towards everyday objects” (Haffenden and Goodale, 2000, 2002).

Further evidence has been provided that visual agnosic patients can show remarkable proficiency when handling common objects in their known surroundings (Bartolomeo et al., 1998; Dijkerman et al., 2004; Karnath et al., 2009); however, to our knowledge, still no systematic investigation has been conducted on the origin of such residual proficiency.

In the present study, we presented a novel case of a patient named AC, with visual agnosia and striking similarities to the previously reported cases of D.F. (Goodale and Milner, 2013; Milner et al., 1991) and J.S. (Karnath et al., 2009). In the recent comprehensive review of 21 agnosic patients, Peel and Chouinard (2023) look for commonalities and unified features of these patients. The most common features were an occipital lesion (20/21), inability to recognize line drawings (19/21), preserved color vision (14/21), and visual field defects (16/21). All these features can be found in AC, which underlines his deficit as visual form agnosia.

We conducted one kinematic experiment to test his reach-and-grasp performance on real objects and investigated his deficits, as well as his residual competencies within the framework of the Two Visual Streams model. We aimed to achieve insights into the nature of those competencies that were spared by the lesion and the functional relationship between the two streams.

Despite significant agnosia for objects, AC could perform everyday activities independently, including cooking, shopping, navigating familiar areas, using public transport, catching a ball, and riding a bike in safe settings (see Tragantzopoulou and Giannouli, 2024 for a critical review on the spatial orientation assessments)^1^. AC displayed a rather preserved color vision, which he mostly relied on while interacting with common-use objects like a pen, a spoon, or a hammer. Indeed, while examining AC, we were intrigued to find him approaching object identification by describing color and texture gradients and focusing on distinctive local details in line with the report on D.F. (Ganel and Goodale, 2019). Thus, to examine such impressive residual proficiency of AC, we designed a grasping experiment based on real-life objects for him. We investigated the effects of masking the color of object stimuli versus presenting them in natural colors and compared immediate grasping responses with delayed ones after prior naming. We assumed that such manipulations at the stimulus and response level would, first, contribute to shedding a better light on the kind and the number of deficits, as well as on the residual competencies of AC, and, second, since they lead to differently weighted activity of the two visual streams (Jax and Buxbaum, 2010; Schenk et al., 2011) they would further contribute to their understanding.

### 1.1 Hypothesis

Regarding the instruction variable, on the one hand, the naming of the objects (i.e., *name-first* conditions) would largely elicit the activation of the VS because of semantic access to object identification (Milner, 2017). On the other hand, reach-and-grasp actions performed immediately after the stimulus onset (i.e., *grasp-first* condition) would minimize further higher-level processing and largely activate the DS (Goodale et al., 1994; Milner et al., 2003). Consequently, since the lesion in AC mostly affected his VS, we hypothesized a larger performance impairment in the name-first condition relative to the grasp-first condition, which instead relied on basically intact DS. For what concerns the object variable, since the color processing along AC’s impaired VS was sufficiently spared (and appeared to be one effective identification cue in everyday life), we hypothesized a detrimental effect of color masking relative to natural color condition, as AC’s VS deficit in object processing would emerge. Possible interactions between instruction and object variables would indicate, for example, whether the VS was mainly in charge of color processing or, as suggested by previous evidence, it also relied on some parts of the DS (Claeys et al., 2004).

## 2 Methods

### 2.1 Case presentation

**Figure 1:**
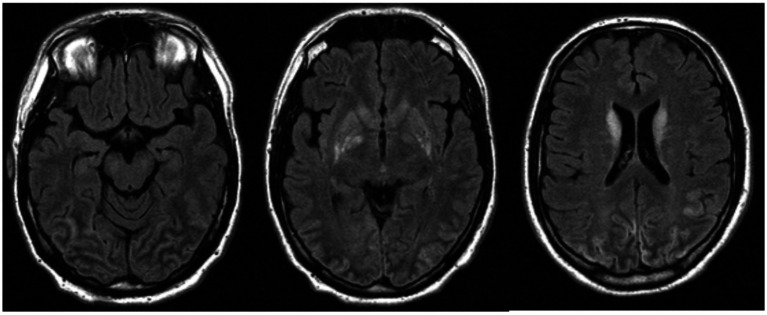
The magnetic resonance image of AC acquired 18 days after the incident, showed edematous alterations and blood–brain barrier dysfunction in the occipital lobes and the posterior-inferior temporal cortex.

In 2010, AC (pseudonym), a previously healthy university student of 29 years, suffered sudden ventricular fibrillation and subsequent cardiac arrest. He received immediate cardiopulmonary resuscitation for a total of about 45 min, followed by intensive care treatment. Initial CT imaging showed no cortical damage or bleeding. Irrespective of severe medical complications (severe inflammatory response syndrome and acute renal failure) within the first week of treatment, AC achieved almost complete physical recovery during the weeks and months that followed. Despite his physical recovery, it became obvious that he still displayed persistent general neuropsychological mild impairment and vision disorders. MR imaging 18 days after the incident showed edematous alterations and blood–brain barrier dysfunction in the occipital lobes and the posterior-inferior temporal cortex (Figure 1). This remained the only MRI of AC’s lesion, as he was later implanted with a cardioverter defibrillator (ICD) to prevent further attacks since medical examinations did not reveal any cause for the initial cardiac arrhythmia. The device was non-MRI-compatible; no further imaging data could be obtained anymore (see the limitations section).

Ophthalmologic examination revealed quadrantanopsia in the left lower quadrant. Moreover, a thorough neuropsychological examination 1.5 years after the incident revealed object agnosia and alexia.

We performed our investigation 5 years after the first neuropsychological examination. We examined the patient using BORB (Birmingham object recognition battery) and VOSP (Visual object and space perception test) (Riddoch and Humphreys, (1993); Warrington and James, 1991). The completion of both tests was due to the severity of the deficit not possible (the details of the neuropsychological examination are presented in a Supplementary File). Additionally, results of the Farnsworth Dichotomous Test for Color Blindness (Panel D-15) (Farnsworth, 1947) revealed adequate color hue discrimination proficiency (1 mistake).

In short, an investigation of the patient’s letter and digit identification and reading skills revealed that he could identify large single letters quite smoothly, as presented in Arial 48. The smaller letters showed AC many more errors. When reading short words, he had to spell them letter by letter, and in some cases, he tracked each letter with his finger, attempting to identify the outline.

Identification of digits were error-prone (e.g., digit 9 mistaken for 0). His own handwriting was small and scrawly but decipherable, although he himself failed to read it afterward. Painting out of memory (e.g., matchstick man, house, sun) was adequate, but lines mostly failed to converge in corners or if he took off the pencil in between. This deficit was mostly due to AC’s difficulty in finding the end of the line drawn so far and his inability to recognize his own drawings. The drawing of presented objects was much better when he could explore them tacitly before. In a test in which AC was asked to trace one of many overlapping lines, he failed because he changed the line at the first crossing. In the FRACT test, AC could not identify the gap even for large Landolt-Cs.

When visually presented with real everyday objects (e.g., cup, corkscrew, lemon, and paper clip), AC drew inferences about the object’s identity out of perceived salient details. When asked to explain his reasoning, he described clues like surface structure (“This is a lemon. I know it because of the surface structure and the color”) or characteristic details (“It’s white, it’s a cup. The handle is located at half past one,” “This has got to be a corkscrew, I recognize it by the wooden handle and the spiral at the center,” “Matchbox, because the sides look somewhat abraded”). Similar part-based recognition approaches have been reported in other cases of visual agnosia, most notably for D.F., who could describe a screwdriver as ‘long, black, thin’ and a pair of scissors as ‘long, thin, silver’ (Milner et al., 1991); D.F. was able to make intelligent guesses, but very slowly, based on such features, whereas objects were swiftly identified by touch (Carey et al., 1996; Milner et al., 1991). However, once an object was presented outside its canonical perspective, AC showed more difficulties (e.g., he identified a pair of scissors only after the blades were closed). For objects that lacked either a characteristic design or when some characteristic clues were masked, he showed a significant deficit in form and identity recognition (apple painted with black color: “It is black, seems to be made of gum or leather, a piece of fruit maybe, I would say it is a ball with a stem, it’s not symmetric”) and sometimes even failed to describe its geometry correctly (large cylindric glass tube: “I would say it is a vase, it is cone-shaped, pointy towards the bottom, it’s open at the top”). In the Odd-One-Out test, where AC was asked to find one different face or object out of five, he could only report the difference when it was marked. Otherwise, he would fail. AC did not demonstrate detectable deficits of spatial orientation. He could navigate easily through the clinic corridors and could go alone to the city and return safely. On a local scale, AC could flowless match the orientation of the right hand to the slot of a rotating disc.

In summary, neuropsychological tests and subsequent investigations suggested that AC’s proficiency in daily interactions with objects was due to knowledge he acquired prior to his disability and his ability to cleverly derive object identity from distinctive visual features. Herein, he particularly relied on color, surface texture, small details, overall size, and positioning in a room (local context) (Details of the neuropsychological assessment are described in Supplementary File 1).

### 2.2 Control participants

A control group of healthy participants was matched to the AC’s age, sex, handedness, and level of education. Participants were recruited in university places; they reported they had no history of neuropsychiatric diseases and no assumption of medications affecting cognitive and motor performance. Thus, 11 male participants (mean age = 30.1, s.d. = ± 2.0, range = 27–33) with ongoing or completed university education (i.e., students from mathematics or engineering university courses) were tested (see Crawford and Garthwaite, 2005 for indications on appropriate group numerosity in single case investigations). They were all right-handed according to the Edinburgh Handedness Inventory (Oldfield, 1971) (mean score = +88.6, s.d. = ± 19.86) and had normal or corrected-to-normal eyesight. The experiment was conducted per the Declaration of Helsinki (1964), and all participants gave their informed consent before testing. The experiment received approval from the ethics committee of UKA (ethics committee approval EK 358–15).

### 2.3 Apparatus, stimuli, and procedure

Participants were seated at a white table. A resting area for the right hand was marked on the table 30 cm to the right of the midsagittal axis, while the left hand was held comfortably underneath the table. A cross in the midsagittal axis 40 cm from the edge of the table marked the place where the objects were presented. A second area, 10 cm from the edge of the table, marked where to place the object after it was grasped.

**Figure 2:**
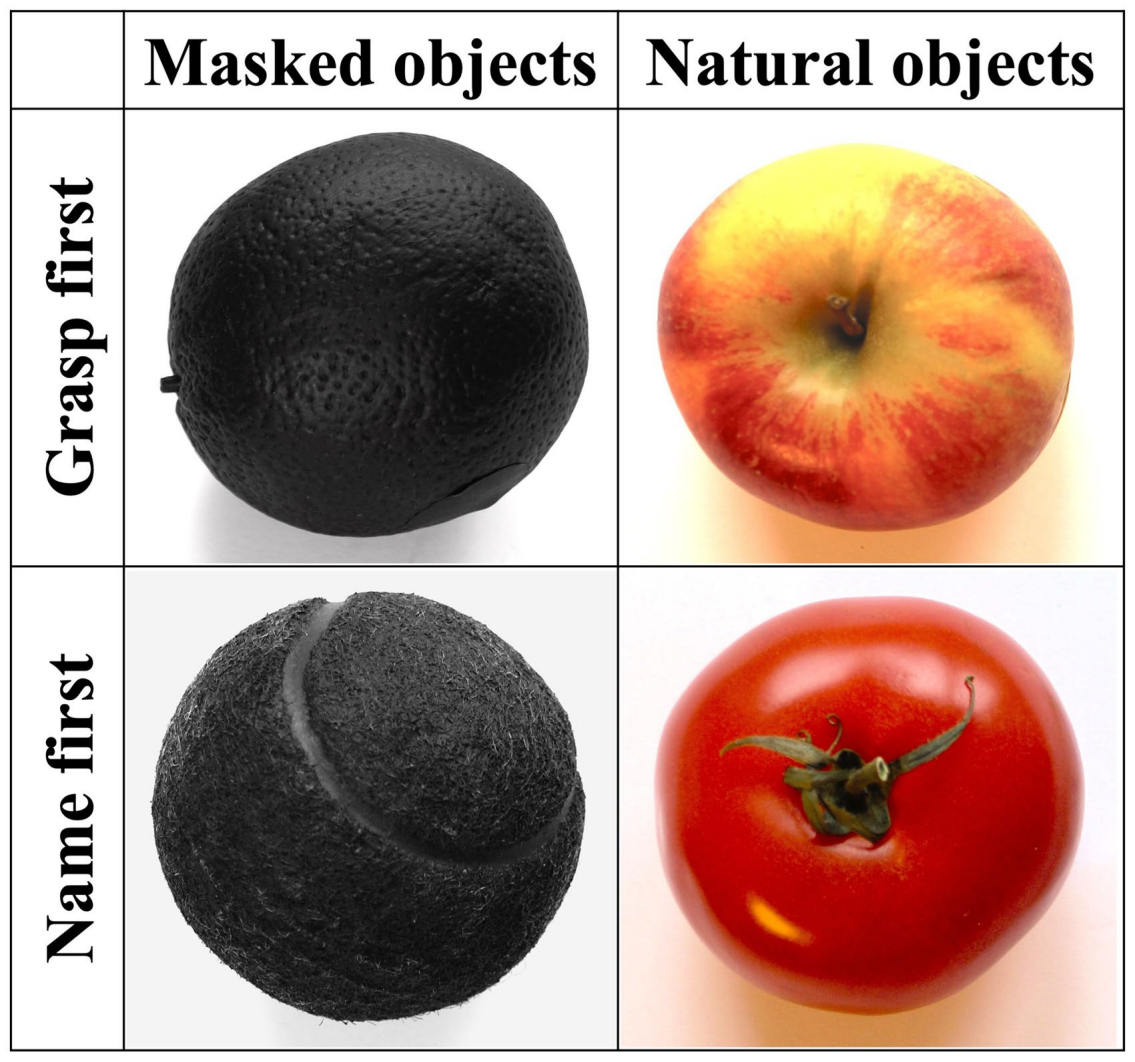
Examples of objects for each of the four experimental conditions (from left to right: orange, apple, tennis ball, tomato).

The experimenter stood in front of the participants, and an opaque barrier prevented them from seeing the to-be-grasped object while the experimenter placed it on the marked cross. Each trial started with the removal of the barrier. Participants (the patient and the healthy control group) were required to grasp with their right hand only. Fifty-two common-use objects with recognizable graspability and stereotypical color, texture, and size were presented once in as many experimental trials. The experimental instructions were verbally given by the experimenter at the beginning of the experiment and before each half of the trial. For the first half of the trials, participants were instructed to grasp the objects as soon as they could see them after the removal of the barrier and place them on the marked area in front of them (i.e., *grasp-first* condition); then, they had to name them aloud. For the second half of the trials, participants first named the objects aloud and then grasped and placed them on the marked area (i.e., *name-first* conditions). This blocked order was adopted to maximize the chances of eliciting selective activation of the dorsal and the ventral stream in the grasp-first and the name-first condition, respectively, avoiding, in particular, possible carry-over activation effects of the ventral stream in the grasp-first trials if preceded by name-first ones. In each grasp., first and name-first condition, half the objects (13) were presented in their original colors (i.e., *natural color* condition), whereas objects from the second half were matched for size and shape to the first half and evenly painted in matt black (i.e., *masked color* condition) while preserving texture and characteristic structural details (see Figure 2). Natural and masked color objects were randomly intermixed with each other within each grasp-first and name-first block and were task-irrelevant.

To observe natural grasping behaviors, participants were not given specific instructions on how to grasp the objects. As a result, we observed pincer grasps for small objects and opposition grasps for larger objects. However, since natural objects can be grasped in variable diameters, instead of examining the absolute maximum grip aperture in mm, we calculated the ratio between the maximum grip aperture and the final grip aperture, that is, when the fingertips touched the object (Borchers and Himmelbach, 2012).

### 2.4 Kinematic data acquisition and pre-processing

Kinematic data were collected using a 3D optical marker-based motion capture system (Vicon Motion systems, Oxford, UK). Fourteen infrared cameras (sampling rate 100 Hz) tracked the motion of small reflective markers (14 mm in diameter) placed on the barrier and the participants’ right hand. Markers were fixed on the tip (center of each fingernail) of the thumb, index finger, middle finger, and ring finger, as well as lateral of the wrist (on the processus styloideus of the os radialis). In addition, we recorded the grasping movements with a video camera (Basler A602fc). Recordings of the grasping movements commenced after a verbal signal by the examiner just before a new trial started and finished when the hand came to rest. For each trial, 3D-Marker positions were reconstructed and labeled (Mthumb, Mindex, Mmiddle, Mring, Mwrist). The VICON System was calibrated each time the measurements were resumed. A stationary and dynamic calibration was performed using a dedicated calibration wand. The spatial resolution of the system was below 1x1x1 mm. Since the kinematic data were acquired by an optoelectronic system that continuously traces the trajectories of the passive markers, interrater variability was not applicable. In order to cope with the noise of the measurement, a low-pass filter was applied to the raw data, with a cutoff frequency of 10 Hz.

All data analyses were performed offline using custom software based on Matlab 2016b (Mathworks Inc., Sherborn, MA, USA). The start of the reaching movement (tstart) was determined when the velocity of the Mwrist exceeded 50 mm/s. The following dependent kinematic parameters of reach-and-grasp action were computed for each trial: movement duration (in ms), Maximum velocity (in m/s), mime of maximum velocity relative to total movement duration (in %), ratio of maximum grip aperture relative to final grip aperture (MGA_ratio; in %), time of maximum grip aperture relative to total movement duration (in %), see also Table A1.

Temporal parameters were determined as relative instead of absolute time values because of the generally decelerated movements performed by the patient relative to the control group. They represented the ratio between the kinematic parameter’s timing and the movement’s total duration.

### 2.5 Statistical analysis

To assess the motor performance of healthy controls, data for each kinematic parameter were submitted to a repeated-measures analysis of variance (ANOVA) performed with SPSS software (IBM SPSS Statistics v. 23) according to a 2 × 2 design: *Object* (natural color vs. masked color) × *Instruction* (grasp-first vs. name-first). Paired-sample *t*-tests were run as post-hoc investigations of significant interactions with Bonferroni-corrected alpha levels for multiple comparisons. The *Singlims* test (Crawford and Garthwaite, 2002) was run to evaluate, on single conditions, whether the patient’s scores met the criterion for a deficit relative to the control group’s scores (e.g., in natural color conditions).

Furthermore, the revised *Tvardiff* test (Revised Standardized Difference Test–RSDT) (Crawford and Garthwaite, 2005) was applied to the difference between the patient’s scores in two conditions (e.g., masked vs. natural color) and the difference provided by the control group between the same conditions [e.g., Patient (masked vs. natural) vs. CG (masked vs. natural)]. In Singlims statistics, we supported the *p*-values with 95% confidence intervals (CIs) for each kinematic measure. We reported the estimated percentage of the normal population falling below an individual’s score and upper and lower limits in square brackets. Since the *Tvardiff* statistics do not compute CIs (Garthwaite and Crawford, 2004), we could not include them in our results.

Since the patient’s performance was expected to be impaired relative to healthy controls at all combinations of object and instruction, no further classification in terms of ‘strong dissociation’ or ‘putatively classical dissociation’ was applicable (Crawford et al., 2010).

Significant statistical outputs resulted in movement duration, maximum velocity, and MGA_ratio kinematic measures.

## 3 Results

### 3.1 Naming accuracy

Patient AC’s object recognition was strongly impaired by color masking. He correctly identified 2 out of 13 masked color objects (15%) with grasp-first instructions and 2 out of 13 masked objects (15%) with name-first instructions. On the contrary, he could correctly name all-natural color objects with both instructions (100%).

The control group (CG) displayed 100% accuracy in all conditions. All healthy participants named the objects swiftly, whereas the patient needed considerably more time (exact naming speeds could not be quantified).

### 3.2 Kinematic performance

#### 3.2.1 Overall effects

Compared to the CG, patient AC displayed considerably longer movement durations (Patient = 1979 ms, CG = 906 ms), Singlims: *t* = 7.125, *p* < 0.001, CI = 100% [100, 100%] and overall lower Maximum velocity (Patient = 447.69 m/s, CG = 781.43 m/s), *t* = 4.699, *p* = 0.001, CI = 0.04% [0, 0.34%]. The patient showed no overall difference compared to the CG regarding the Time of maximum velocity, MGA_ratio, and Time of MGA, Singlims: *t*s < 1.

#### 3.2.2 Effect of object

The CG showed slightly longer movement duration and lower Maximum velocity with *masked* objects relative to *natural* objects (967 ms *vs.* 926 ms, 756.44 m/s *vs.* 785.79 m/s), *F*(1,10) = 8.243, *p* = 0.017, ƞ^2^_p_ = 0.452; *F*(1,10) = 7.009, *p* = 0.024, ƞ^2^_p_ = 0.412.

**Figure 3:**
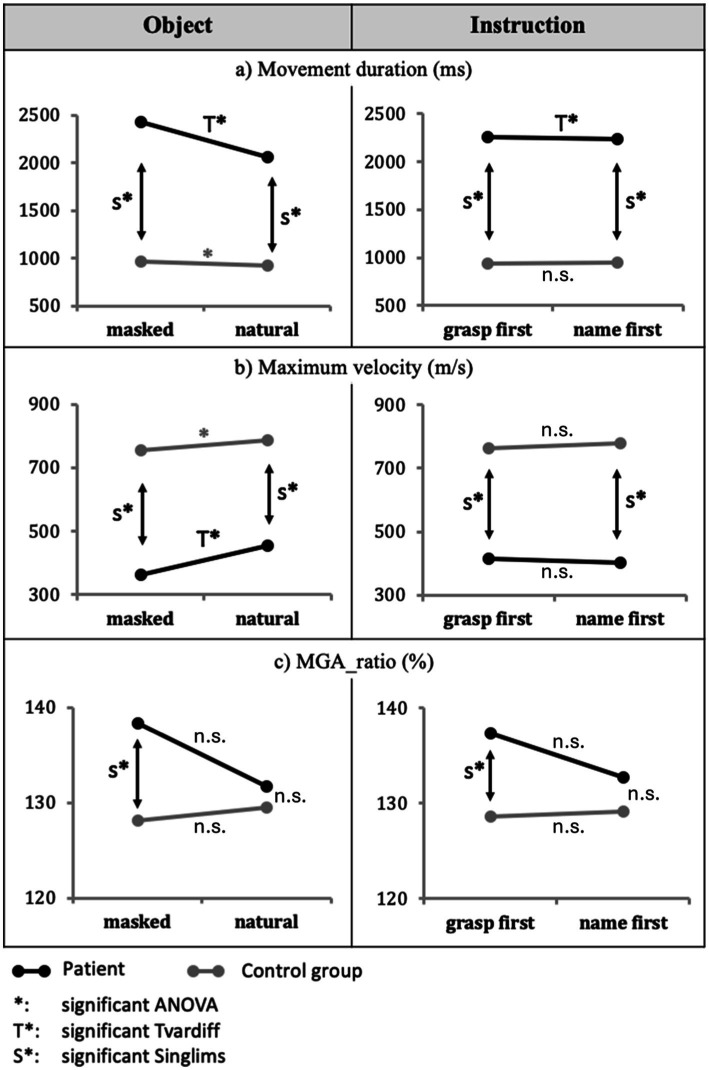
Main effects of object and instruction. The effects of the *object* (masked vs. natural) for the Patient AC and the control group regarding **(A)** movement duration, **(B)** Maximum velocity, and **(C)** MGA_ratio are displayed in the left column. Results for *instruction* (grasp-first vs. name-first) are displayed in the right column. Significant differences are indicated with * for the CG and T* for patient AC. Moreover, significant differences in Crawford’s Singlims comparison between the patient and the CG are indicated with S*. Non-significant differences are indicated with n.s.

Compared to the CG, the patient displayed much longer movement duration and lower Maximum velocity with *masked* relative to *natural* objects (2,433 ms *vs.* 2065 ms), *t*(10) = 6.304, *p* < 0.001 (Figure 3A, T* in left panel); (363.01 m/s *vs.* 454.21 m/s), *t*(10) = 2.866, *p* = 0.017 (Figure 3B, T* in left panel). Furthermore (and consistent with overall differences), when comparing the patient and the CG within object conditions, the patient showed in both masked and natural objects significantly longer movement duration and lower maximum velocity than the CG: Movement duration with masked objects *t*(10) = 9.853, *p* < 0.001, CI = 100% [100, 100%] and with natural objects *t*(10) = 7.280, *p* < 0.001, CI = 100% [100, 100%] (Figure 3A, S* in left panel), and Maximum velocity with masked objects *t*(10) = −5.231, *p* < 0.001, CI = 0.02% [0.00, 0.12%] and with natural objects *t*(10) = −4.273, *p* < 0.001, CI = 0.08% [0.00, 0.73%] (Figure 3B, S* in left panel).

Regarding the hand aperture measures, the CG displayed no significant difference between masked and natural conditions for MGA_ratio, *F*(1,10) = 1.293, *p* = 0.282, ƞ^2^_p_ = 0.115; so did the patient relative to the CG, *t*(10) = 1.385, *p* = 0.196 (Figure 3C, left panel).

The CG displayed later MGA_time with *masked objects* relative to *natural objects* (76% vs. 74%), *F*(1,10) = 8.289, *p* = 0.016, ƞ^2^_p_ = 0.453, whereas the patient displayed a non-significant difference relative to the CG (*masked objects* = 76.5%, *natural objects* = 79%), *t*(10) = 1.406, *p* = 0.190.

#### 3.2.3 Effect of instruction

The CG showed no significant main effect of instruction in movement duration, *F*(1,10) = 0.030, *p* = 0.866, ƞ^2^_p_ = 0.003, Maximum velocity, *F*(1,10) = 0.870, *p* = 0.373, ƞ^2^_p_ = 0.08, and in any other kinematic dependent variable, *F*s(1,10) < 1.327, *p*s > 0.05 (Figure 3, right panels).

The patient displayed longer movement durations in the *grasp-first* condition compared to the *name-first* condition (grasp-first = 2,256 ms, name-first = 2,242 ms), *t*(10) = 4.093, *p* = 0.002 (Figure 3A, right panel).

#### 3.2.4 Effect of object × instruction interaction

**Figure 4:**
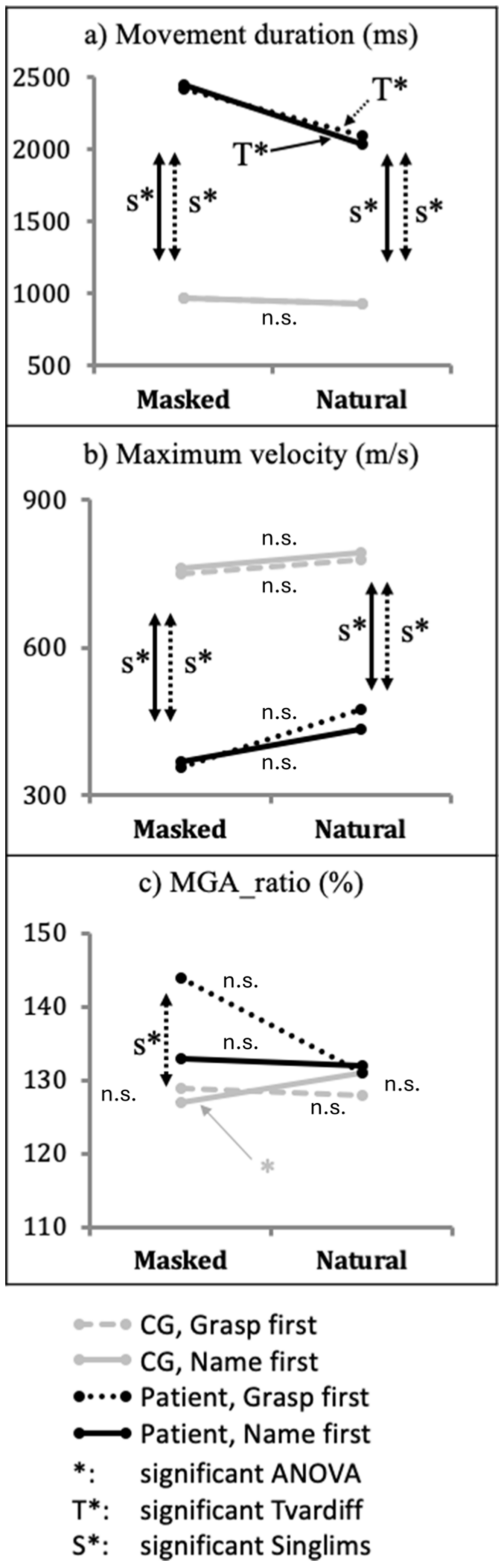
Effect of object × instruction interaction. Performance differences of patient AC (black lines) and Control group (gray lines) between objects (masked vs. natural) at each instruction (grasp-first vs. name-first) for the dependent variables: **(A)** *movement duration*, **(B)** *maximum velocity,* and **(C)** *MGA_ratio*. Significant differences are indicated with * for the CG and T* for patient AC. Moreover, significant differences in Crawford’s Singlims comparison between the patient and the CG are indicated with S*. Non-significant differences are indicated with n.s.

In the CG, we observed a non-significant object × instruction interaction for movement duration, *F*(1,10) = 0.002, *p* = 0.965, ƞ^2^_p_ = 0.0002 (Figure 4A, gray and gray dotted lines). The patient AC showed longer movement duration with *masked* than with *natural* objects in both *grasp-first* conditions, *t*(10) = 7.921, *p* < 0.001, and *name-first* condition, *t*(10) = 2.983, *p* = 0.014. Moreover, the patient displayed a shorter movement duration for *natural* objects in *name-first* (2036 ms) relative to *grasp-first* condition (2095 ms), *t*(10) = 2.412, *p* = 0.037. For *masked* objects, the trend was opposite but non-significant (*name-first* = 2,449 ms, *grasp-first* = 2,417 ms), *t*(10) = 2.001, *p* = 0.073 (Figure 4A, black and black dotted lines).

For MGA_ratio, we observed a significant interaction in the CG, *F*(1,10) = 7.428, *p* = 0.021, ƞ^2^_p_ = 0.43. Post-hoc paired-samples *t*-tests showed that for *name-first* conditions, the CG displayed larger MGA_ratio with *natural* compared to *masked* objects, *t*(10) = − 2.428, *p* = 0.036, while for *grasp-first* condition, the CG showed no difference between objects, *t*(10) < 1, *p* = 0.401. Moreover, for *natural* objects, the CG showed a larger MGA_ratio in *name-first* relative to *grasp-first* conditions (*name-first* = 131%, *grasp-first* = 128%), *t*(10) = −2.516, *p* = 0.031. For *masked* objects, the difference was not significant (*name-first* = 127%, *grasp-first* = 129%), *t*(10) = 1.724, *p* = 0.115 (Figure 4C, gray and gray dotted lines).

Compared to the CG, the patient showed a larger MGA_ratio when he grasped *masked* objects with *grasp-first* instructions, *t* = 2.481, *p* = 0.016, CI = 98.38% [90.56, 99.99%]. The patient showed no significant difference in all other conditions relative to the CG, Singlims *t*s < 1.537 (Figure 4C, S*).

## 4 Discussion

In our study, we compared the grasping of naturalistic and color-masked objects, which were either named first and grasped afterward or grasped first and named afterward, inpatient AC with visual agnosia, and a group of healthy control participants.

Our patient AC demonstrated a damaged location associated with a pattern of impaired processing and residual competencies consistent with several recently reviewed agnosia patients, including DF (Peel and Chouinard, 2023). In synthesis, AC’s damage affected the ventral visual stream. His inability to identify common-use objects most likely reflected a processing problem associated with the synthesis of several visual discontinuities in brightness, textures, or colors into complex stimuli (see Goodale and Milner, 2013 for a review). His residual abilities allowed him to access the gross shape on the one hand and the small details of objects, but he could not perform any further integration of such separate features into one perceptual representation, which ultimately led to recognition. Since real, tangible objects offer several identity cues like color and surface texture, AC could take advantage of such information to make inferences about the identity of perceived objects. Our experiment demonstrated that AC’s preserved color vision and processing were utilized to maximize the accuracy of object identification.

Specifically, masking the color of objects had a slight influence on the performance of the healthy control group: it slowed grasping actions despite flawless verbal identification. This further supported the evidence that in healthy populations, color information is a sensitive cue for object identification (Mapelli and Behrmann, 1997; Bramão et al., 2011; Chouinard and Goodale, 2012). Healthy participants displayed no difference between immediate, swift grasping (i.e., grasp-first) and prior visual identification of the target objects (i.e., name-first).

In contrast, patient AC’s performance was characterized by significantly larger effects. He failed to identify most of the color-masked objects and demonstrated an even longer movement duration, lowering maximum velocity towards these objects. Regarding hand aperture, AC showed no overall impairment of MGA-ratio compared to the CG. However, when grasping masked objects under time constraints (grasp the first condition, see Figure 4C), AC displayed a significantly larger MGA ratio than the CG. Conversely, with natural objects that provided ample color information, AC performance with respect to hand aperture ratio was comparable to healthy controls.

In the following, we discuss these results in relation to the Two Visual Stream hypothesis and other known cases of visual agnosic patients.

### 4.1 Object identification

The healthy participants were able to identify all objects without errors, irrespective of masking, in both grasp-first and name-first conditions.

In the experiment, AC was able to identify 100% of the naturally colored objects, but after marked delay. This indicated his impressive remaining object identification skills with regard to everyday objects. It has been previously shown that visually agnosic individuals perform well with everyday objects (Dijkerman et al., 2004; Goodale and Milner, 2013; Karnath et al., 2009). Moreover, evidence has been provided by another visual agnosic patient who better recognized highly familiar objects before the brain damage occurred (Rennig et al., 2018). While identifying objects, beyond taking advantage of their informative visual details, AC openly reported considering color information; indeed, he focused on color hues and gradients to identify natural objects. This compensatory strategy resulted in abnormally prolonged naming latencies and is consistent with previous evidence of better recognition, within visual agnosia deficits, for real objects in pictures compared to line drawings (Mapelli and Behrmann, 1997). Similar detail-based recognition approaches and delays in naming tasks have also been described in other visual agnosic patients, for example, D.F. (Humphrey et al., 1994; see also Peel and Chouinard, 2023). In contrast, AC was able to identify only 15% of color-masked common objects, thus supporting the notion that his object identification skills relied on color information for applying the aforementioned color- and detail-based approach.

In the cortex, color and form information is hierarchically processed across multiple levels from the retina to visual areas V1 to V4 and higher-level VS regions (Conway, 2009; Seymour et al., 2009). Although color and form representations are anatomically co-localized and highly interactive, they remain segregated along the VS (Lafer-Sousa et al., 2016; Taylor and Xu, 2022). In particular, visual area V4 is regarded as a mid-level processing stage along the VS that facilitates color perception, texture perception, and shape processing and is strongly interconnected with many other cortical areas (Felleman and Van Essen, 1991; Pasupathy et al., 2020). Beyond V4, the representation of color and shape are more segregated and can be selectively damaged, while the other functions remain preserved (Bouvier and Engel, 2005; Taylor and Xu, 2022). At these higher-level cortical areas, the conscious association of color and shape (e.g., when identifying objects by color) can also be selectively damaged (Siuda-Krzywicka and Bartolomeo, 2020; Stasenko et al., 2014). The well-preserved color processing of AC, as demonstrated in his good scoring on the Farnsworth Dichotomous Test (Farnsworth, 1947), indicates that the areas for association of color and object shape were preserved. More in detail, AC plausibly retained functionality of lower- and mid-level visual areas, including V4, so that he could code color and texture. AC’s preserved color vision is in line with previous evidence of other visual agnosia patients, like D.F.: Color vision and the conscious association of color and shape improved her voluntary interaction with objects (Dijkerman et al., 2004; Goodale and Milner, 2013). Neuroimaging studies demonstrated some remaining VS activation when she was presented with colored images (James et al., 2003). Neuroimaging studies also detected separate areas for the processing of texture, color, and shape and demonstrated compromised activation of D.F’s shape discrimination area, while activation in her texture and color discrimination areas was spared (Cavina-Pratesi et al., 2010).

The consistency of our data with previous clinical evidence suggests that visual agnosic patients skillfully use their remaining color vision in combination with detail-based recognition approaches in everyday activities. This plausibly contributes to their surprisingly proficient handling of common objects.

### 4.2 Kinematic analysis

Healthy participants were assessed to establish baseline performance in the grasping task. Color masking did not affect the visual identification of objects (100% correct for both masked and natural objects). With respect to kinematic performance, the masking of color had a small effect: movement duration was slightly longer (Figure 3A, left panel, grey line), and Maximum velocity was reduced (Figure 3B, left panel, gray line) relative to natural color. No main effect of instruction (i.e., grasp-first vs. name-first) was observed (Figure 3, right panel). Thus, the baseline task of grasping everyday objects did not represent a problem in individuals with normal visual performance, except for some slight effects of color-masking.

The Two Visual Stream model posits that the immediate grasping of objects is subserved by the DS, whereas their visual identification is processed in the VS (Milner and Goodale, 2008). This model further postulates a gradual processing shift from one stream to the other (Milner and Goodale, 2006); namely, an increasing contribution of the VS would depend on the increasing amount of visual identification processing.

Our results on the healthy control group fit well within this model, suggesting that normal performance with color-masked objects requires additional processing load along the VS because of unavailable color information. This ultimately increased movement duration and reduced maximum velocity relative to natural color objects.

Interestingly, performance stayed detrimental with masked relative to natural colors irrespective of whether the objects were gasped immediately or grasped after being named. This suggests that the lack of color information also posed challenges to immediate grasp-DS processing (for which color cues within objects should not be essential to perform visuomotor transformations) and is consistent with the view that color information could also be processed along the healthy DS when color is behaviorally relevant (Claeys et al., 2004; Toth and Assad, 2002). Animal studies show that parts of area V4 show strong connections with dorsal stream areas such as DP, VIP LIP, PIP, or MST. This could be the direct link between VS and DS, providing color information to areas processing action, spatial vision, and spatial attention (Roe et al., 2012; Baizer et al., 1991; Ungerleider et al., 2008).

The CG showed no overall effect for maximum grasp aperture. Familiarity with objects has previously been shown to improve grasp scaling compared to grasping neutral cuboids (Borchers and Himmelbach, 2012). Moreover, maintaining characteristics like texture and surface pattern, e.g., roughness, has previously been shown to improve accurate grasp scaling (Haffenden and Goodale, 2002). Healthy individuals displayed a significant interaction between color and instruction for MGA-ratio (Figure 4C, gray lines). It indicated the most accurate hand aperture accommodation when objects’ color was masked, and reach-and-grasp actions started after their naming (i.e., name-first). This could also be explained by the additional delay that healthy control persons displayed when naming masked objects and by an increased contribution of the VS to the programming of reach-and-grasp actions.

All in all, we would argue that healthy participants were able to maintain accurate maximum grasp aperture towards highly familiar but color-masked objects by slowing their grasping performance, thereby allowing increased VS processing. Such basic evidence regarding the CG is essential to interpret AC’s performance.

In visual agnosia patients like AC, VS processing is impaired, albeit partially spared (Goodale and Milner, 2018; James et al., 2003). The VS provided AC with proficient visual identification of natural-colored objects but impaired verbal identification of masked objects (only 15%). Compared to healthy individuals, AC displayed much longer movement duration and lower Maximum velocity with masked objects relative to natural objects; this was evident in both grasp-first and name-first conditions (Figures 4A,B, black lines). These findings indicate that visual processing in AC’s impaired VS was affected by color masking to a larger extent than the CG, irrespective of the amount of semantic processing prior to grasping. Thus, the object identification process (which was challenged in the CG), resulting in corruption in AC due to his lesioned VS. This effect persisted in both VS- and DS-supported grasping conditions. As for the CG, the detrimental effect of color-masking on grasping performance in grasp-first conditions might be explained in the wake of previous evidence that color information would also be processed in the early stages of the DS (Claeys et al., 2004; Roe et al., 2012; Baizer et al., 1991; Ungerleider et al., 2008) and that VS and DS would interact when handling known objects (Milner, 2017).

In contrast to the CG, such an effect was also evident in AC with natural objects. With natural objects, AC’s movement duration was longer (and maximum velocity was lower - even if not significant) in the grasp-first condition relative to the name-first condition. This suggests that time constraints and minimized semantic processing (grasp-first conditions) impaired his performance even with natural-color objects. This is well in line with his VS impairment.

To complete the picture, the patient’s MGA ratio was not different from that of the CG for *natural* objects irrespective of grasp-first and name-first instructions and for *masked* objects in name-first condition (Figure 4C, black lines). This is consistent with the view that *natural* objects provided sufficient color and texture information to the patient’s VS in the name-first condition to facilitate accurate grasp scaling like in the CG. We would argue that the slowing of AC’s performance facilitated comparably skillful grasp scaling with natural objects in grasp-first conditions. Conversely, with *masked* objects in grasp-first conditions, AC showed increased MGA-ratio (Figure 4C, dotted lines). We suggest that under time constraints and with reduced color information, the patient’s damaged VS could not contribute sufficiently to his DS programming, thus leading to a safety grasp (i.e., an increased MGA ratio).

All in all, these results bear several interesting implications for motor interaction with highly familiar objects. In the visual agnosic patient AC, grasping performance was significantly slower when objects were color-masked. This delay presumably allows the activation and the processing of color and semantic information in the patient’s damaged VS. This adaptation allowed rather unimpaired performance regarding his gasp aperture. Interestingly, the slowing effect of color-masking was also observed in the immediate grasping (grasp-first) condition. Immediate grasping is commonly understood to be facilitated by the fast-processing DS (Milner and Goodale, 2008). This surprising sensitivity of the DS to color-masking would be in line with previous evidence of color processing in some parts of the DS (Claeys et al., 2004). Thus, the slowing of movement performance allowed the patient’s grasp scaling to remain intact. Indeed, his grasp scaling only differed from healthy participants in immediate grasping (grasp-first) and with color-masked objects. According to the two visual stream models, immediate grasping should be facilitated by the DS. The failure of the patient to achieve proficient grasp scaling underlines the importance of collaboration between the two visual streams for skillful interaction with everyday objects.

Moreover, our results suggest that individuals with visual agnosia can facilitate grasping actions to a level comparable to healthy individuals, even in immediate response situations, if ample visual information is provided. This plausibly explains the slowed and deliberate object interaction that visual agnosic patients show in their everyday surroundings, which we have observed in AC and which has also been reported for other visual agnosic patients (Bartolomeo et al., 1998; Goodale and Milner, 2013). In a kinematic study of patient DF’s reach-and-grasp performance, Whitwell et al. (2015) provided evidence of a significant role of terminal tactile feedback and real-time visual information in keeping the dorsal visuomotor system operating normally for prehensile acts. Analogously, our study suggests that color information also plays a similar role.

## 5 Limitations

The patient AC was reported to us 5 years after the first neuropsychological examinations that were performed in sub-acute clinical conditions. Given his initial severe clinical conditions and the later implantation of a non-MRI-compatible cardioverter defibrillator (ICD), it has been impossible to send the patient to further clinical and research investigations involving imaging sessions. Thus, the only available MR imaging dated 18 days after the incident illustrates a sub-acute scenario with edematous alterations beyond lesion areas.

Typically, brain edema develops in the acute stage of ischemia and is much larger than the actual lesion. However, even if it is relatively reabsorbed after some time, in the areas surrounding the lesion, there can be selective neuronal death so that these tissues can be disturbed in their function as well. For these reasons, AC’s deficit is best described in clinical terms.

Moreover, after he resigned from the hospital, AC declared himself unavailable for further follow-up imaging examinations, including high-resolution CT scans compatible with his ICD.

Limitations apply to interpretations of the kinematic findings and of their relationship with the underlying neurological impairment since no assessment of cognitive functions beyond the visuo-perceptual domain was performed.

Control samples in single-case studies in cognitive neuropsychology are typically modest, so N < 10 is not unusual, and Ns < 20 are very common (Crawford and Howell, 1998; Crawford and Garthwaite, 2005; Whitwell et al., 2015). Assuming this, we cannot entirely rule out the possibility that our N = 11 sample may be a limitation to the extent of our statistical inferences.

## 6 Conclusion

In the present study, we performed a kinematic investigation on one individual with visual agnosia. While integrating his clinical evidence with the existing and most representative cases, we further contributed to the understanding of visual information processing through the well-established Two Visual Stream model.

We put in evidence the crucial role of color information in compensating identification and motor programming deficits. Preserved color processing in V4 allowed the VS, as well as the DS, to utilize color information to interact with objects effectively.

Overall, we supported the view that visual agnosia provides a valuable model for studying the adaptive use and flexible interaction of visual streams based on task demands.

## Data Availability

The original contributions presented in the study are included in the article/Supplementary material. Further inquiries can be directed to the corresponding author/s.

## Footnotes

^1^Spatial orientation abilities were self-reported by AC relative to those activities he used to conduct in his own surroundings or were directly observed by the clinicians in the hospital setting. No dedicated and systematic neuropsychological assessments were conducted by the medical team during AC’s acute phase and his subsequent rehabilitation phase. For a recent critical review of currently available spatial navigation tests and the employment of cutting-edge VR-based assessment tools, see Tragantzopoulou and Giannouli (2024).

## Appendix

**Table A1:** Operationalization of the kinematic parameters.

Parameter	Abbreviation	Unit	Description
Movement duration	MD	S	tend – tstart
Maximum velocity	vmax	mm/s	The peak of Mwrist path velocity between tstart and tend
Relative time of maximum velocity	%timev max	%	Moment of vmax relative to MD
Maximum hand aperture	aperturemax	Mm	Maximum linear 3D distance between Mthumb and the selected opponent finger marker from tstart to tend
Final hand aperture	aperturefinal	Mm	Linear 3D distance between Mthumb and the selected opponent finger marker at tend
Ratio of maximum vs. final hand aperture	apertureratio	%	aperturemax / aperturefinal * 100

Temporal parameters were determined as relative instead of absolute time values because of the generally decelerated movement displayed by the patient. Statistically significant results were obtained for movement duration, maximum velocity, and MGA_ratio measures.
