# Supplementary material for: Differential affection of the visual information sub-streams in a patient with visual agnosia
Source: Front Psychol. 2025 Feb 12;16:1452979. doi: 10.3389/fpsyg.2025.1452979 (PMC11862234; doi:10.3389/fpsyg.2025.1452979)
Supplement: Supplementary file 1 [file Data_Sheet_1.docx]

**Differential affection of the visual information sub-streams in a patient with visual agnosia**

***Supplementary materials***

Lederer K^1^, Fimm B^2^, Munzert J^3^, Reiser M^3^, Maurer H^3^, Binkofski F^1,4,5*^ Pellicano A^6*^

1. Division for Clinical Cognitive Research, Department of Neurology, University Hospital RWTH Aachen, Aachen, Germany
2. Department of Neurology, University Hospital RWTH Aachen, Aachen, Germany
3. Institute for Sport Sciences, University of Gießen, Gießen, Germany
4. Institute for Neuroscience and Medicine (INM-4), Research Center Jülich GmbH, Jülich, Germany
5. JARA Brain
6. Department of Educational Sciences, University of Catania, Catania (Italy)

^*^ Shared senior authorship

Corresponding author:

Prof. Dr. med. Ferdinand Binkofski

Institute for Neuroscience and Medicine (INM-4)

Research Center Juelich GmbH,

Wilhelm-Jonen-Strasse,

52428 Juelich, Germany

**Neuropsychological testing:**

A complete neuropsychological profile was not possible, because of the severe visual deficit of AC. The main focus of the testing was visuo-perceptive assessment.

Test battery for the assessment of attention:

Visual field test:

Incomplete anopsia in the left lower quadrant.

Active visual field:

The task was to react to flanker stimuli and to detect changes in brightness in different parts of the visual field. AC had marked difficulties to master this test. Only 15 out of 25 changes of brightness in the peripheral visual field could be detected. Marked differences in the visual fields could be recognized with the exception of the left lower visual field.

Farnsworth Test for color perception:

Normal result.

Cambridge Low Contrast Grating:

Contrast sensitivity left eye = 120, right eye = 88. Mean binocular CS was 310.9±249.0 cps.

Letter Naming:

Only letter A (presented in Arial) was recognized. Letters H, R, Z could not be recognized; AC could not trace these letters with his finger or to draw them. As a rule, vertical lines were recognized as first, but some letters were illusionary misrecognized, for example, letter H was traced as letter R; Z was traced as E.

Furthermore, reading of words was tested. With the word “Apfel” (apple), AC recognized first A, then L and finally F. Only after a long period of trying could AC work out the correct word “Apfel”.

In the word “Auto” (car), only the letter A could be recognized. When asked how many letters there were between the first and the last, AC correctly stated two. A four digits number was identified as a word. After a while, AC guessed that it could be a number. However, AC misread “2” as “5” and “5” was named as “3 or 8”; AC failed to identify further digits.

The letter “M” could be drawn from memory but could not be read and recognized later.

Recognition of faces:

Faces of well-known personalities were presented in color or black-white photos. The faces could be recognised as such, and the eyes were the main stimulus that facilitated their identification. In most cases AC could tell whether the face presented was female or male. AC could not detect if a photo was presented upside down. While he could still locate the eyes, he erroneously presumed the nose and the mouth to be located below the eyes.

Naming of objects:

When presented with real objects, AC correctly identified only a few of them. He described a trumpet as “metallic”. Presented with a battery, AC described it on the first attempt as a “small can”, “round”, “red-white-blue” or as “metallic”. As soon as the battery was put upright, he could recognize it as “battery”. AC failed to recognize a pair of scissors when they were presented with the blades open. However, when presented with the blades closed, AC succeeded in identifying them. In sum, as soon as the canonic position of an object was abandoned, AC’s recognition became markedly poorer.

When presented with a matchbox, AC first described it as “a rectangle”, “like a deck of carts”, “blue-red on the top”, or “cassette”. After the matchbox was presented with its abrasive side clearly visible, AC recognized it as a matchbox through it being “worn off on this sides”. A key was described by AC as “brass colored” and then several details were named (“a hole in the middle”, “round”, “it has a style on the top”). He then continued to speculate about its identity: “bottle opener” and named further details: “the style has a certain structure on the top”. After tactile exploration, AC could identify the key, immediately. After being presented with an array of real objects, he identified and named several features of them and derived their identity out of these attributes. Again, in some cases AC succeeded in recognizing the real objects only after tactile exploration.

Thus, this appeared to be the peculiar approach adopted by AC to identify real objects.

Odd-one-out:

Several drawings of objects or faces were presented in one row, and AC was instructed to find out the one (object or face) which was different from the others.

AC succeeded in detecting only those odd objects or faces, which had very prominent differences to the rest. However, if an object differed only in one detail, the odd one was not recognized at all.

Line tracing:

An image of several tangled lines with various intersections was presented. AC was instructed to trace one line from start to end while ignoring the other lines. At the first crossing, AC took the wrong line to continue his trail. This suggested that AC failed to find a structure in the composition of lines.

FRACT-Test for the assessment of visual acuity and contrast perception:

Even for large Landolt-Cs which were presented on a screen, AC could not find and indicate the characteristic gap.

Naming of Snodgrass-Pictures, faces and line drawings:

We obtained quite a clear indication that AC depended on single features (i.e., color, elements, form) when he reconstructed objects from his memory. This was only possible when the features gave unequivocal clues to the identity of the object. For example, a banana was recognized because it was bent.

Perceptual orientation matching task (Goodale et al., 1991):

AC was able to match errorless the orientation of the rotated slot.

Card-posting and perceptual orientation matching task (Goodale et al., 1991):

AC was able to match errorless the orientation of the rotated slot, as well as to post the card into it.

Goodale, M. A., Milner, A. D., Jakobson, L. S., & Carey, D. P. (1991). A neurological dissociation between perceiving objects and grasping them. Nature, 349(6305), 154–156. <https://doi.org/10.1038/349154a0>
